# Machine learning for the prediction of mortality in patients with sepsis-associated acute kidney injury: a systematic review and meta-analysis

**DOI:** 10.1186/s12879-024-10380-6

**Published:** 2024-12-21

**Authors:** Xiangui Lv, Daiqiang Liu, Xinwei Chen, Lvlin Chen, Xiaohui Wang, Xiaomei Xu, Lin Chen, Chao Huang

**Affiliations:** 1 Department of Intensive Care Medicine, Affiliated Hospital of Chengdu University, Chengdu, Sichuan China; 2 Department of Nursing, Affiliated Hospital of Chengdu University, Chengdu, Sichuan China; 3 Chengdu University, Chengdu, Sichuan China

**Keywords:** Machine learning, Sepsis-associated acute kidney injury, Mortality, Meta-analysis

## Abstract

**Background:**

Predicting mortality in sepsis-related acute kidney injury facilitates early data-driven treatment decisions. Machine learning is predicting mortality in S-AKI in a growing number of studies. Therefore, we conducted this systematic review and meta-analysis to investigate the predictive value of machine learning for mortality in patients with septic acute kidney injury.

**Methods:**

The PubMed, Web of Science, Cochrane Library and Embase databases were searched up to 20 July 2024 This was supplemented by a manual search of study references and review articles. Data were analysed using STATA 14.0 software. The risk of bias in the prediction model was assessed using the Predictive Model Risk of Bias Assessment Tool.

**Results:**

A total of 8 studies were included, with a total of 53 predictive models and 17 machine learning algorithms used. Meta-analysis using a random effects model showed that the overall C index in the training set was 0.81 (95% CI: 0.78–0.84), sensitivity was 0.39 (0.32–0.47), and specificity was 0.92 (95% CI: 0.89–0.95). The overall C-index in the validation set was 0.73 (95% CI: 0.71–0.74), sensitivity was 0.54 (95% CI: 0.48–0.60) and specificity was 0.90 (95% CI: 0.88–0.91). The results showed that the machine learning algorithms had a good performance in predicting sepsis-related acute kidney injury death prediction.

**Conclusion:**

Machine learning has been shown to be an effective tool for predicting sepsis-associated acute kidney injury deaths, which has important implications for enhancing risk assessment and clinical decision-making to improve sepsis patient care. It is also eagerly anticipated that future research efforts will incorporate larger sample sizes and multi-centre studies to more intensively examine the external validation of these models in different patient populations, allowing for a more in-depth exploration of sepsis-associated acute kidney injury in terms of accurate diagnostic efficacy across a diverse range of model and predictor types.

**Trial registration:**

This study was registered with PROSPERO (CRD42024569420).

**Supplementary Information:**

The online version contains supplementary material available at 10.1186/s12879-024-10380-6.

## Introduction

Sepsis is a life-threatening organ dysfunction due to a dysregulated host response to infection [1]. Sepsis-associated acute kidney injury (S-AKI) is one of the most common organ dysfunctions in hospitalised and critically ill patients [2]. S-AKI accounts for 45-70% of AKI cases in the ICU, with a mortality rate of 60-80% [3, 4]. Sepsis is one of the leading causes of death globally, affecting more than 19 million people each year, so S-AKI is an important public health issue [5, 6]. In the early stages of AKI, there are no obvious clinical symptoms, and the diagnosis of AKI is based on a decrease in renal function (increased creatinine or decreased urine output), but the diagnosis of AKI is usually delayed until the actual renal function is abnormal [7, 8]. Although some novel biomarkers such as blood and urine can identify AKIs at an early stage [9, 10]. However, the diagnostic reliability of individual biomarkers is imprecise and lacks tissue correlation. It is well known that there is no effective treatment for AKI other than renal replacement therapy. Early identification of high-risk individuals and effective intervention can help improve the prognosis and survival of S-AKI patients [11, 12].

Machine learning can use big data analytics to predict future events and can help clinicians make accurate diagnoses and treatments [13]. Machine learning is capable of handling complex, large amounts of health data and is widely used in the construction of clinical predictive models [14]. Recent studies have shown that machine learning algorithms achieve better performance in predicting S-AKI prognosis [15–17]. However, there is a deficiency of systematic evidence on the prediction of S-AKI mortality risk. Therefore, we conducted this systematic review and meta-analysis to assess the predictive value of machine learning for S-AKI mortality risk and to provide guidance for the development and updating of S-AKI mortality risk prediction tools.

## Methods

The study protocol was registered with the international prospective systematic evaluation registry PROSPERO (CRD42024569420) and was conducted in accordance with the standard guidelines provided by the Preferred Reporting Items for Systematic Reviews and Meta-Analyses PRISMA-2020.

### Literature search

Systematic literature searches were performed using PubMed, Web of Science, Cochrane Library and Embase. Our search strategy used a combination of subject terms and free words. We limited the search language to English. Two researchers independently searched the literature (Xiangui Lv and Xinwei Chen), and any disagreements were resolved by a third researcher (Chao Huang).The details of the search are given in Additional fle 2. The main search terms included machine learning, sepsis, acute kidney injury, and death.20 July 2024 was the last date of the search.

### Inclusion and exclusion criteria

## Inclusion criteria

(1) Study subjects aged ≥ 18 years; (2) Study subjects with sepsis; (3) Study designs included cohort studies, case-control studies, and cross-sectional studies; (4) The outcome event predicted by the model was mortality, a machine learning prediction model was constructed, and the process of model building, validation, and evaluation was described.

## Exclusion criteria

(1) Risk factor studies only, without complete risk modelling; (2) Case series, case reports, randomised controlled trials and descriptive surveys; (3) Guidelines, expert opinions, reviews and animal studies; (4) Language: other than English; (5) Inaccessibility of original text or incomplete data.

## Literature screening and data extraction

After the literature search was completed, it was imported into EndNote X9 for management. Literature was screened by 2 researchers (Xiangui Lv and Chao Huang) by reading the title, abstract and other information of the literature and strictly following the inclusion and exclusion criteria in order to select eligible studies. If the 2 researchers did not agree on the final inclusion of literature, a 3rd senior researcher (Daiqiang Liu) was sought for judgement and agreement.

## Data extraction

After confirming the inclusion of the literature, download and read the original article in its entirety. A table was generated to record all relevant data. All extracted data items were collected according to the Cochrane guidance for data collection and the Critical Appraisal and Data Extraction for Systematic Reviews of Prediction Modelling Studies (CHARMS) checklist [18], including year of publication, type of study, country, data sources, Patient characteristics, AKI diagnostic criteria, Study Outcome, Delete missing variables, Missing variables imputation method, Predictor selection, machine learning algorithms, type of validation, Calibration indicators and so on. The primary outcomes included in the study were C index, sensitivity and specificity.

### Risk of Bias Assessment

The Prediction Model Risk of Bias Assessment Tool (PROBAST) was used to evaluate the risk of bias in the included studies [18]. PROBAST includes two parts: risk of bias evaluation and applicability evaluation. The risk of bias evaluation is conducted in four areas: Participants, Predictors, Outcome, and Analysis, and each area contains 2/3/6/9 landmark questions. The evaluation of applicability was conducted in 3 domains: participants, predictors, and outcomes. The evaluation of the risk of bias of all the included literature was done independently by 2 researchers (Daiqiang Liu and Chen Xinwei), and the disagreement was discussed and resolved by negotiation, and if there was still disagreement, the decision was arbitrated by the third researcher (Huang Chao).

### Statistical analysis

A meta-analysis of the evaluation metrics (C-index, sensitivity and specificity) of the machine learning models was performed by applying STATA 14.0 software. If the C-index did not have a 95% CI or SE, the SE was estimated using the calculation proposed by Debray et al. [19]. The random effects model was preferred in the meta-analysis of the C-index given the presence of different variables and parameter inconsistencies in the learning model. In addition, we used bivariate mixed-effects models for sensitivity and specificity meta-analyses.

## Results

### Study selection

**Fig. 1:**
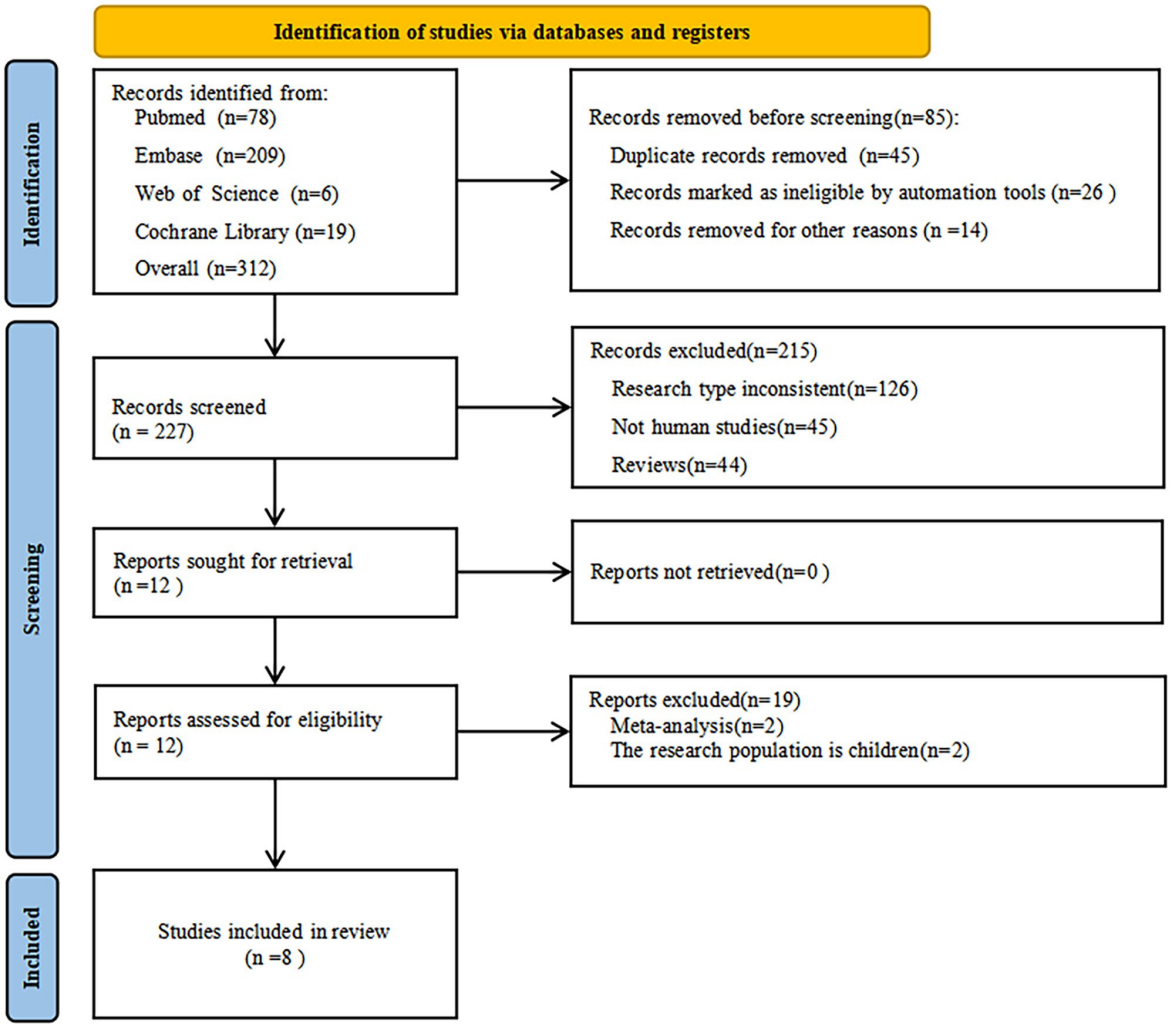
PRISMA study selection flowing chart

A total of 312 publications were searched through various databases, including PubMed (*n* = 78), Embase (*n* = 209), Web of Science (*n* = 6), and Cochrane Library (*n* = 19). Through a cascade of screening, eight papers met the inclusion criteria and were included in our study, Fig. 1 shows the PRISMA flowchart.

### Study characteristics

The 8 studies were published between 2022 and 2024, with investigators from China and were retrospective cohort studies. A total of 59,094 patients were enrolled across five studies. Data were obtained from public databases such as MIMIC-IV, MIMIC-III, eICU and hospital electronic medical record data. Seven studies included adult patients aged 18 years and older [15, 16, 20–24], One study included elderly patients aged 65 years or older [17]. Five studies focused on in-hospital mortality outcomes [15–17, 21, 24], three studies focused on mortality at 28 days after admission to ICU [20, 22, 23]. Detailed characteristics of the included studies are shown in Table 1.

**Table 1:** Characteristic of the included studies

Author	Year	Study design	Country	Data sources	Disease background	AKI diagnostic criteria	Study outcome
Lei Dong [15]	2024	Retrospective cohort	China	MIMIC-IV、MIMIC-III、Beijing Friendship Hospital affiliated with Capital Medical University	Sepsis patients aged 18–89 in ICU	2012 KDIGO	In-hospital mortality
Zhiyan Fan [20]	2023	Retrospective cohort	China	MIMIC-IV, Hangzhou First People’s Hospital Affiliated to Zhejiang University School of Medicine	Sepsis patients over 18 years old in ICU	2012 KDIGO	28d mortality
Tianyun Gao [16]	2024	Retrospective cohort	China	MIMIC-IV	Sepsis patients over 18 years old in ICU	2012 KDIGO	In-hospital mortality
Xunliang Li [21]	2023	Retrospective cohort	China	MIMIC-IV	Adult patients with sepsis who developed AKI within 48 h after ICU admission.	2012 KDIGO	In-hospital mortality
Xiaoqin Luo [22]	2022	Retrospective cohort	China	MIMIC-IV、eICU	Adult patients with sepsis who developed AKI within 48 h after ICU admission.	2012 KDIGO	28d mortality
Jie Tang [17]	2024	Retrospective cohort	China	MIMIC-IV	Sepsis patients over 65 years old in ICU	2012 KDIGO	In-hospital mortality rate
Jijun Yang [23]	2023	Retrospective cohort	China	MIMIC-IV	Sepsis patients over 18 years old in ICU	2012 KDIGO	28 day mortality
Hongshan Zhou [24]	2023	Retrospective cohort	China	MIMIC-IV、Xiangya Hospital of Central South University and Xiangya Third Hospital of Central South University	Sepsis patients over 18 years old in ICU	2012 KDIGO	In-hospital mortality

S-AKI, Sepsis-associated acute kidney injury; NA, Not Applicable

### Features of machine learning Algorithme

In the included studies, all authors used a variety of different machine learning methods to construct multiple predictive models. These algorithms are then compared to determine the machine learning algorithm that performs best. A total of 53 models were constructed in 8 studies, and 17 machine learning algorithms were used, including Random Forest (RF), Extreme Gradient Boost (XGBoost), Logistic Regression (LR), Support Vector Machine (SVM), K Nearest Neighbor (KNN),Multilayer perceptron(MLP), Naive Bayes(NB), Adaptive Boosting(AdaBoost), Categorical Boosting(CatBoost), Decision Tree(DT), Gradient Boosting Machines(GBM), Neural Network(ANN), Gradient Boosting Decision Tree(GBDT), Light Gradient Boosting Machine (LightGBM), Rpart, Support Vector Classifer(SVC), Least absolute shrinkage and selection operator(LASSO).

**Fig. 2:**
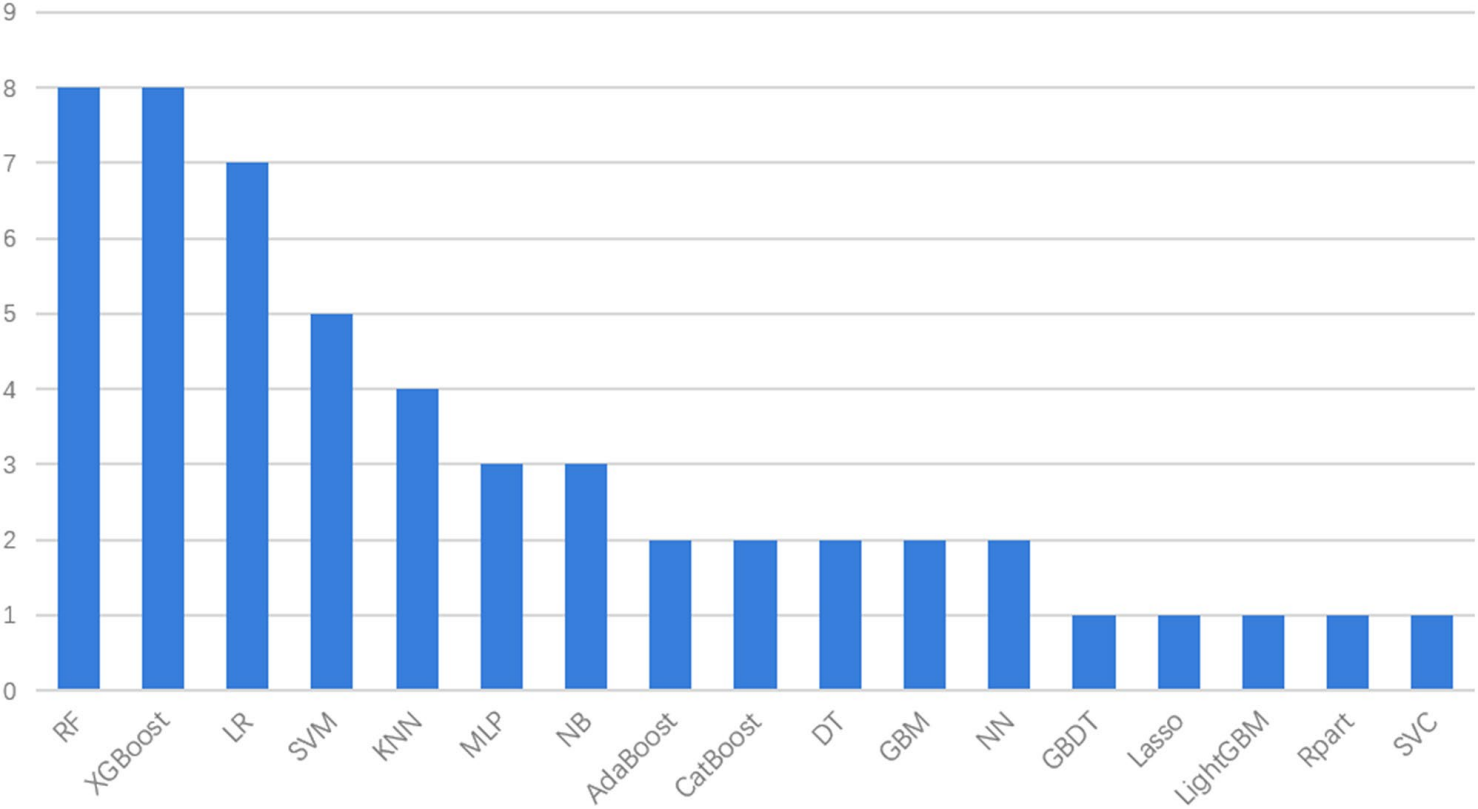
Distribution of machine learning algorithms

Regarding the types of machine learning algorithms, RF and XGBoost had the highest frequency (*n* = 8), followed by LR (*n* = 7), SVM (*n* = 5), and KNN (*n* = 4). Distribution of machine learning algorithms used in the 8 studies is shown in Fig. 2. XGBoost showed the best predictive performance in 5 studies [15, 20–23]. CatBoost showed the best predictive performance in 2 studies [17, 24]. RF showed the best predictive performance in 1 study [16]. However, choosing the right machine learning algorithm cannot completely determine the performance of the model, because the performance of the model may also be affected by the choice of predictors, hyperparameters and other factors [25]. The model has adopted different interpolation methods such as XGBoost, MiceForest, and KNN in handling missing data. Fan et al. adopted the Reduced Feature Elimination (RFE) algorithm to discover the key predictive factors of their machine learning model [17, 20, 24]. Logistic Regression [15], Lasso Regression [15, 21], Random Forest [15], XGBoost [22], and Boruta algorithms [23] are used to select the most important predictive factors for predicting S-AKI mortality. There were 3 studies with internal and external validation [15, 20, 24], 5 study with internal validation [16, 17, 21–23]. The characteristics of the machine learning model are shown in Table 2. Machine learning model performance, including C-index, accuracy, sensitivity, specificity, and F1 score, was used to assess and characterise model performance. Detailed information on model performance is provided in Additional fle 3. The C-index ranged from 0.574 to 0.987 and performed well in most studies.

**Table 2:** Machine learning model features

Author	Age (years)	Death/S-AKI cases	Machine learning algorithme	Best Algorithm	Delete missing variables	Imputation method	Predictor selection	Validation	Calibration indicators
Lei Dong	NA	1769/7544	LR, Lasso, Rpart, RF, XGBoost, and ANN	XGBoost	>30%	KNN	LR, Lasso, and RF	Bootstrap and external validation	Calibration curve, Brier score, and kappa coefficient
Zhiyan Fan	NA	NA/2599	LR, RF, XGBoost, MLP, and SVC	XGBoost	>30%	MiceForest	Recursive feature elimination	Random sampling and external validation	NA
Tianyun Gao	67.0 ± 16.1	2352/12,196	KNN, XGBoost, NB, DT, SVM, linear/rbf), RF, and LR	RF	>25%	MiceForest	RF	10-fold cross-validation	Calibration curve
Xunliang Li	68.7(57.2,79.6)	1629/8129	LR, SVM, KNN, DT, RF, and XGBoost	XGBoost	NA	MiceForest	LASSO	Random sampling	Calibration curve
Xiaoqin Luo	69 (58,79)	NA/9537	XGBoost, RF, and SVM	XGBoost	NA	XGBoost	XGBoost	Random sampling	Calibration curve
Jie Tang	77 (71, 84)	1813/6613	LR, SVM, GBM, AdaBoost, XGBoost、CatBoost, NB, NN, MLP, KNN, and RF	CatBoost	>5%	KNN	Recursive feature elimination	Random sampling	Calibration plot
Jijun Yang	67(57,78)	1940/9158	LR, RF, GBM, and XGBoost	XGBoost	>20%	RF	Boruta algorithm	5-fold cross validation	Calibration curve
Hongshan Zhou	67.7 ± 15.2	16,154/3318	KNN, AdaBoost, MLP, SVM, LR, NB, GBDT, RF, LightGBM, and XGBoost	CatBoost	NA	KNN	Recursive feature elimination	Random sampling and external validation	NA

### Quality of evidence and risk of bias

**Fig. 3:**
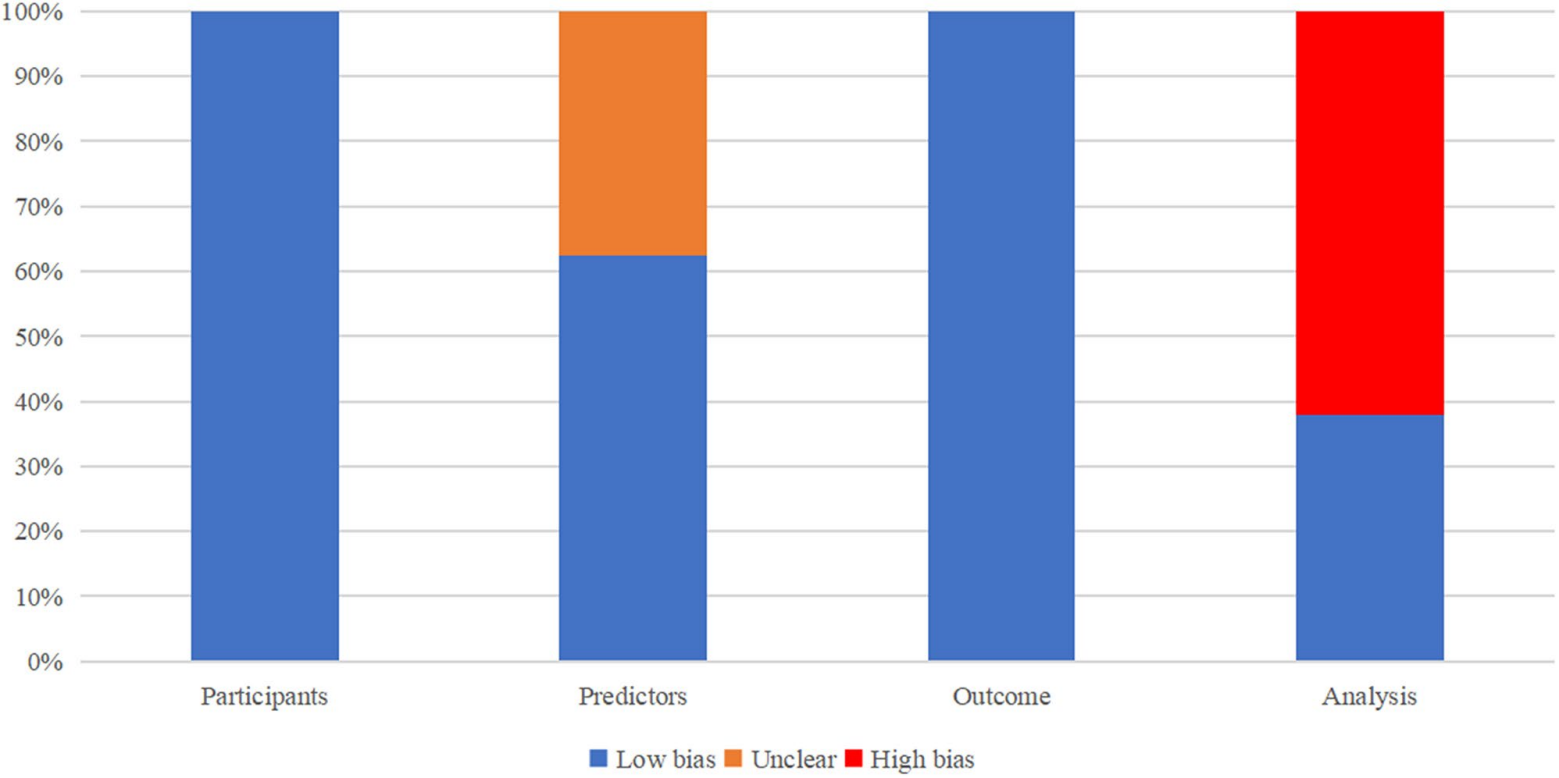
Risk of bias assessment

The risk of bias assessment was carried out independently by two evaluators (Xiangui Lv and Xinwei Chen), and any discrepancies were resolved by a third evaluator (Chao Huang). The PROBAST assessment tool was used to evaluate the risk of bias of the prediction models. The assessment was conducted in four domains: participants, predictors, outcome and analysis. 4 studies were considered to be at high risk of bias in the predictors domain [15, 22–24], which may be attributed to the fact that the retrospective study data were obtained from a multicentre clinical database and not using a unified method to evaluate predictive factors. In the evaluation of outcomes, due to the specificity of the outcome indicator of death, the results of the evaluations related to the definition of outcomes in the included studies were all at low risk of bias. The inclusion of the model validation set is mostly generated through random sampling for internal validation, with only three multicenter studies having independent external validation datasets. The risk of bias assessment of the included studies is shown below in Fig. 3.

### Statistical analysis

**Fig. 4:**
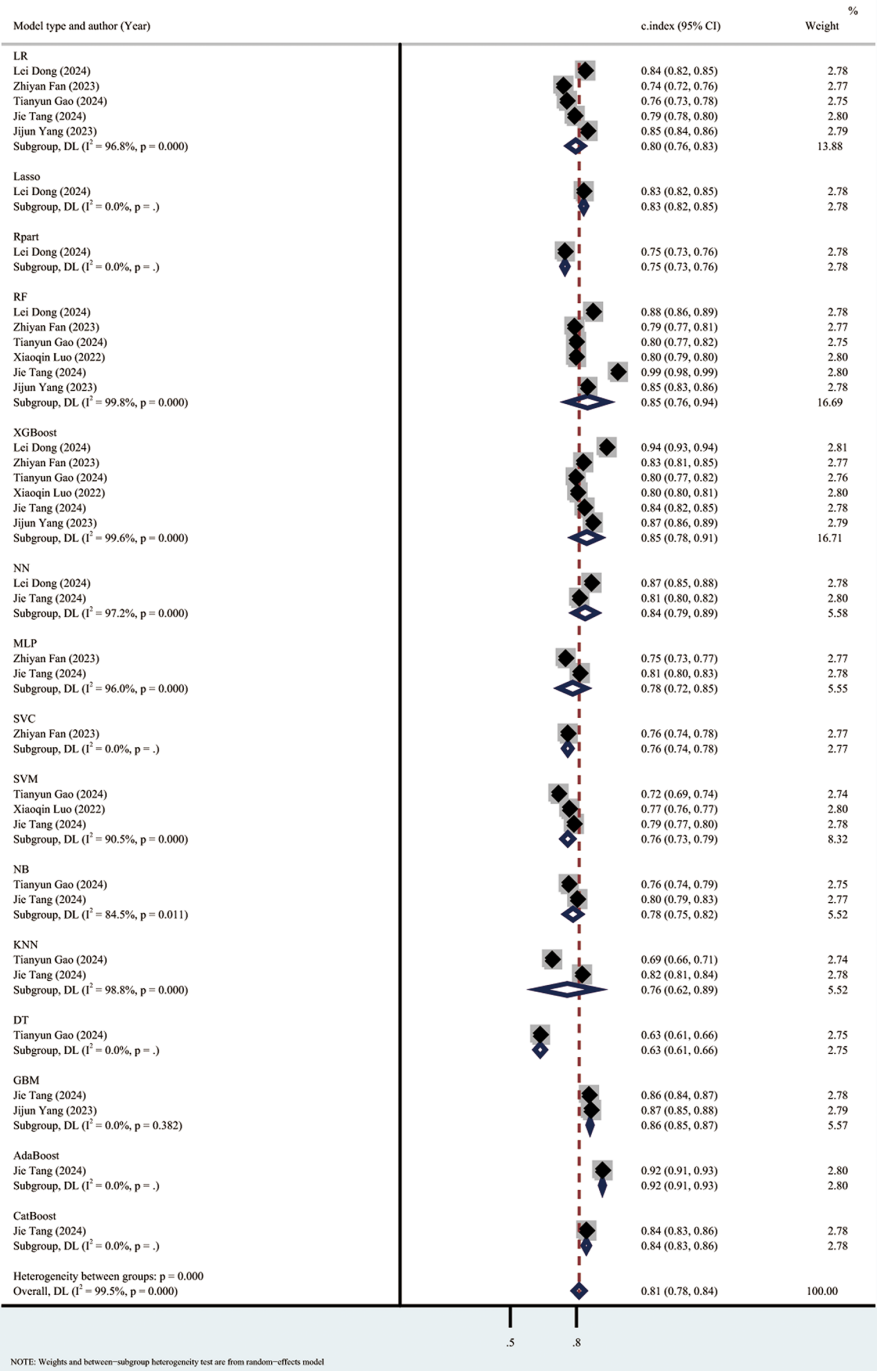
Forest plot of C-index meta-analysis of prediction models for S-AKI death prediction in the training set

**Fig. 5:**
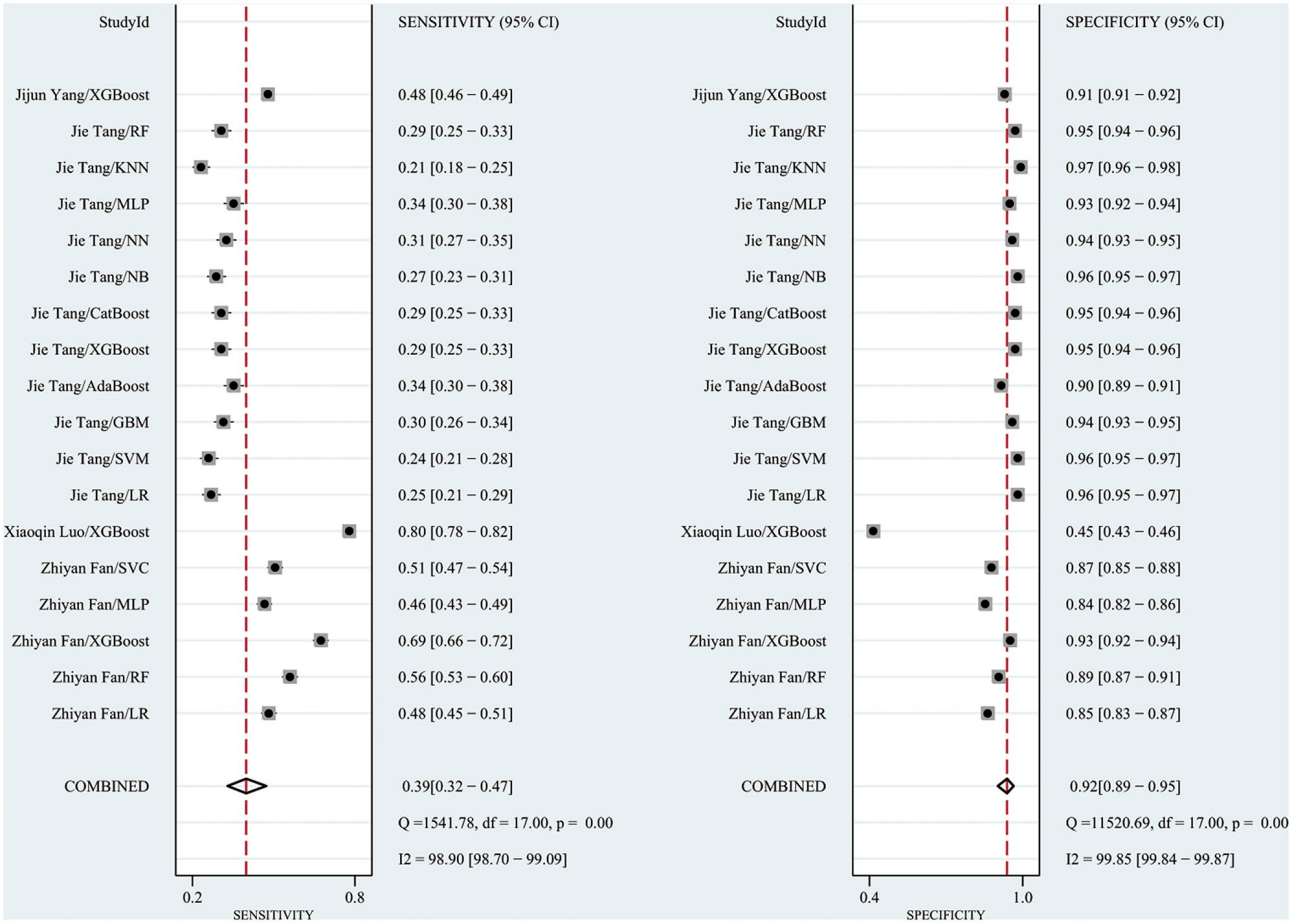
The results of the sensitivity-specific meta-analysis in the training set

The meta-analysis using the random effects model showed that the C index in the training set was 0.81 (95% CI: 0.78–0.84), the sensitivity was 0.39 (0.32–0.47), and the specificity was 0.92 (95% CI: 0.89–0.95). The subgroup analyses of the C index in the training set were 0.80 (95% CI: 0.76–0.83) for the LR model, 0.85 (95% CI: 0.76–0.94) for the RF model, 0.85 (95% CI 0.78, 0.91) for the XGBoost model, and 0.76 (95% CI 0.73, 0.79) for the SVM model. (Forest plot of c-index meta-analysis of prediction models for S-AKI death prediction in the training set are shown in Fig. 4, and the results of the sensitivity-specific meta-analysis in the training set are shown in Fig. 5).

**Fig. 6:**
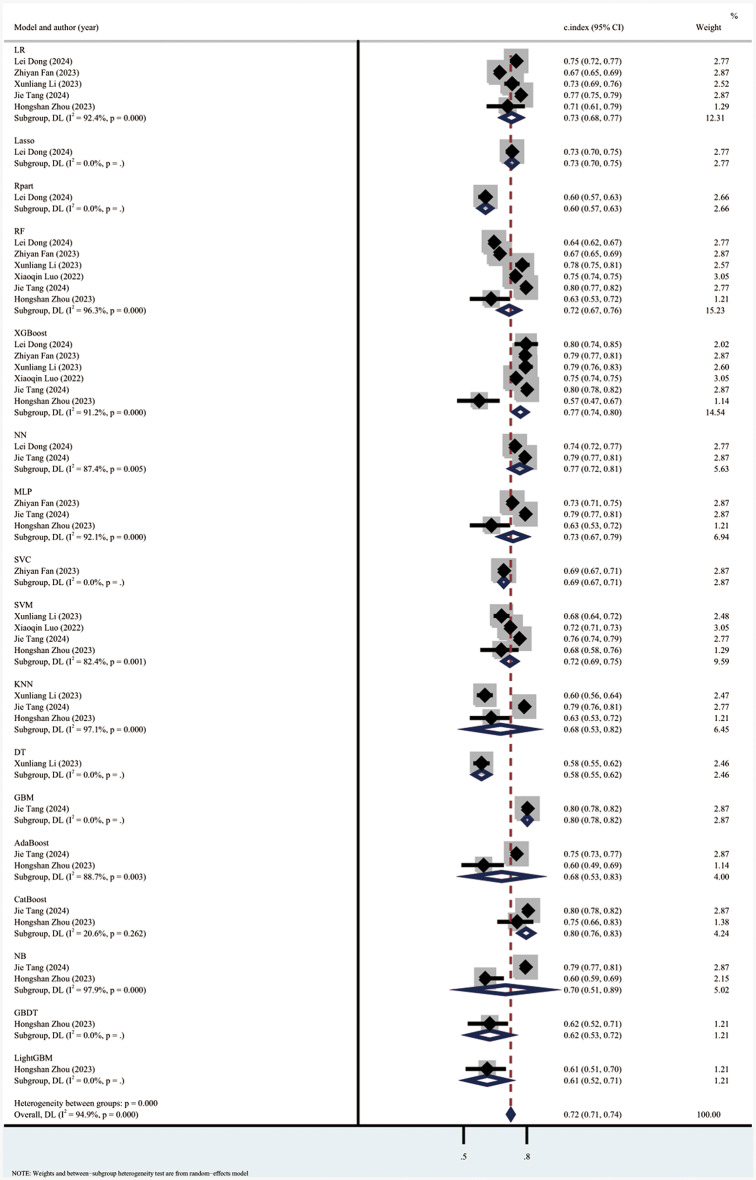
Forest plot of C-index meta-analysis of prediction models in the validation set for the prediction of S-AKI deaths

**Fig. 7:**
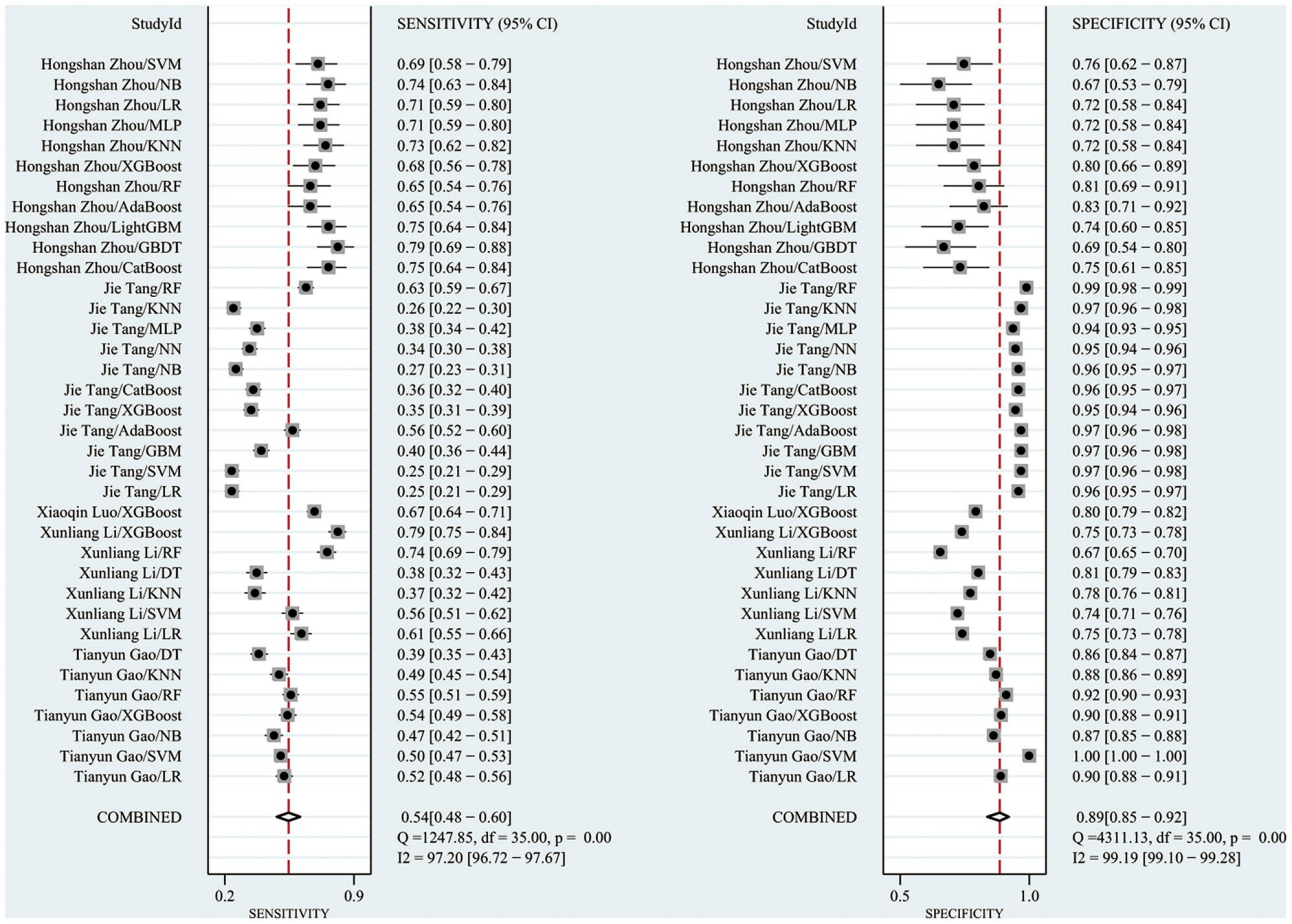
The results of the sensitivity-specific meta-analysis in the validation set

The C index in the validation set was 0.73 (95% CI: 0.71–0.74), with a sensitivity of 0.54 (95% CI: 0.48–0.60) and a specificity of 0.90 (95% CI: 0.88–0.91). The subgroup analyses of the C-index of the validation set were 0.74 (95% CI: 0.70–0.78) for the LR model, 0.73 (95% CI: 0.69–0.78) for the RF model, 0.78 (95% CI 0.75, 0.81) for the XGBoost model, 0.73 (95% CI 0.67, 0.79) for the MLP model, and 0.71 (95% CI 0.67, 0.79) for the SVM model was 0.71 (95% CI 0.69, 0.74), NB model was 0.73 (95% CI 0.66, 0.80), and KNN model was 0.68 (95% CI 0.59, 0.77). The results showed that the machine learning models had good performance in predicting sepsis-related acute kidney injury death prediction. (Forest plot of c-index meta-analysis of prediction models in the validation set for the prediction of S-AKI deaths are shown in Fig. 6, and the results of meta-analysis of the sensitivity specificity in the validation set are shown in Fig. 7).

## Discussion

This study is a systematic review evaluating the predictive potential of machine learning models for predicting the risk of death in S-AKI. 53 prognostic prediction models from 8 studies were investigated, and our survey incorporated 17 different machine learning Algorithms, including RF, XGBoost, LR, SVM, KNN, MLP, NB, AdaBoost, CatBoost, DT, GBM, ANN, GBDT, LightGBM, Rpart, SVC, and LASSO. This systematic evaluation and meta-analysis highlights the invaluable role of machine learning models in predicting the risk of death in patients with S-AKI, and that these predictive models exhibit good predictive performance, supported by high c-index values in both the training and validation sets. The C index was 0.81 (95% CI: 0.78–0.84) in the training set and 0.73 (95% CI: 0.71–0.74) in the validation set. In addition, the training set sensitivity was 0.39 (0.32–0.47) and specificity was 0.92 (95% CI: 0.89–0.95). The validation set sensitivity was 0.54 (95% CI: 0.48–0.60) and specificity was 0.90 (95% CI: 0.88–0.91). The results of this study suggest that machine learning performs well in predicting the risk of death from S-AKI and can be used as a potential tool for predicting the risk of death from sepsis-related acute kidney injury. This has important implications for clinicians to enhance risk assessment and clinical decision making to improve S-AKI patient care.

RF, XGBoost, and LR are the top three popular machine learning algorithms for S-AKI mortality risk prediction. The C-index of the XGBoost model was 0.85 (95% CI 0.78, 0.91) in the training set and 0.78 (95% CI 0.75, 0.81) in the validation set. XGBoost, as a machine learning technique, is optimised for speed and scalability, making it one of the most efficient gradient boosting algorithms available. It is efficient and flexible in dealing with missing data, and is able to assemble weak predictive models to construct accurate predictive models [26]. Due to its excellent accuracy values and performance, XGBoost has been considered the best algorithm for machine learning and prediction competitions and is widely used to predict adverse clinical outcomes [27].Therefore, we propose to advance the development of XGBoost-based predictive models for use in a wide range of diseases. Based on our findings, machine learning has demonstrated excellent predictive capabilities in sepsis-associated acute kidney injury, further highlighting its potential to improve patient care and outcomes.

## Conclusion

This systematic evaluation and meta-analysis examined the valuable role of machine learning models in predicting the risk of death in S-AKI patients. The results show that machine learning has good performance in identifying the risk of death in S-AKI, but its predictive accuracy still needs to be improved. This has important implications for enhancing risk assessment and clinical decision making to improve sepsis patient care. In our study, we found that the XGBoost model exhibited the best predictive performance and was most commonly used to predict the risk of sepsis-related acute kidney injury death. We also eagerly look forward to incorporating larger sample sizes and multicentre studies in future research efforts to more deeply examine these models for external validation in different patient populations, which will allow us to explore the precise diagnostic effects of S-AKI, across a variety of model and predictor types, in more depth.

### Limitations

Undoubtedly, our study is not without limitations. Firstly, despite our comprehensive search, the number of included studies was still small, probably due to our focus on English language publications. Second, the diversity of included models led to heterogeneity, and the different variables included in the models may be some of the sources of the heterogeneity observed in our study. Again, most of the studies in this review used MMIC and eICU datasets to develop and evaluate models. Future research should use external datasets to validate machine learning models to ensure their stability and applicability in a wider population. Finally, this study focused on the accuracy of machine learning in predicting the risk of death from S-AKI and did not include risk factors that contribute to death from acute kidney injury in sepsis.

## Electronic supplementary material

Below is the link to the electronic supplementary material.

Supplementary Material 1: PRISMA checklist

Supplementary Material 2: Literature search strategy

Supplementary Material 3: Detailed information on model performance

## Data Availability

The datasets used and/or analyzed during the current study are available from the corresponding author on reasonable request.
