# Supplementary material for: Machine learning for the prediction of mortality in patients with sepsis-associated acute kidney injury: a systematic review and meta-analysis
Source: BMC Infect Dis. 2024 Dec 21;24:1454. doi: 10.1186/s12879-024-10380-6 (PMC11663330; doi:10.1186/s12879-024-10380-6)
Supplement: Supplementary file 2 — Supplementary Material 2: Literature search strategy [file 12879_2024_10380_MOESM2_ESM.docx]

Table S1 Literature search strategy

**1.Pubmed**

| Search number | Query | Results |
| --- | --- | --- |
| #1 | "Machine Learning"[MeSH Terms] | 74,573 |
| #2 | "machine learning"[Title/Abstract] OR "deep learning"[Title/Abstract] OR "transfer learning"[Title/Abstract] OR "prediction model"[Title/Abstract] OR "artificial intelligence"[Title/Abstract] OR "artificial neural network"[Title/Abstract] OR "support vector machine"[Title/Abstract] OR "random forest"[Title/Abstract] OR "XGboost"[Title/Abstract] | 266,557 |
| #3 | #1 OR #2 | 274,464 |
| #4 | "Sepsis"[MeSH Terms] | 147,337 |
| #5 | "Sepsis"[Title/Abstract] OR "bloodstream infection"[Title/Abstract] OR "infection bloodstream"[Title/Abstract] OR "Septicemia"[Title/Abstract] OR "Septicemias"[Title/Abstract] OR "blood poisoning"[Title/Abstract] OR "blood poisonings"[Title/Abstract] OR "poisoning blood"[Title/Abstract] OR "severe sepsis"[Title/Abstract] OR "sepsis severe"[Title/Abstract] OR "Pyemia"[Title/Abstract] | 152,099 |
| #6 | #4 OR #5 | 233,170 |
| #7 | "Acute Kidney Injury"[MeSH Terms] | 59,022 |
| #8 | "acute kidney injury"[Title/Abstract] OR "kidney injuries acute"[Title/Abstract] OR "kidney injury acute"[Title/Abstract] OR "acute renal injury"[Title/Abstract] OR "acute renal injuries"[Title/Abstract] OR "kidney failure acute"[Title/Abstract] OR "acute kidney failures"[Title/Abstract] OR (("Kidney"[MeSH Terms] OR "Kidney"[All Fields] OR "kidneys"[All Fields] OR "kidney s"[All Fields]) AND "failures acute"[Title/Abstract]) OR "acute kidney failure"[Title/Abstract] OR "acute renal failure"[Title/Abstract] OR "renal insufficiency acute"[Title/Abstract] OR "acute renal insufficiencies"[Title/Abstract] OR (("renal insufficiency"[MeSH Terms] OR ("Renal"[All Fields] AND "Insufficiency"[All Fields]) OR "renal insufficiency"[All Fields] OR ("Renal"[All Fields] AND "Insufficiencies"[All Fields]) OR "renal insufficiencies"[All Fields]) AND "Acute"[Title/Abstract]) OR "acute kidney insufficiency"[Title/Abstract] OR "acute renal insufficiency"[Title/Abstract] OR (("Kidney"[MeSH Terms] OR "Kidney"[All Fields] OR "kidneys"[All Fields] OR "kidney s"[All Fields]) AND "insufficiency acute"[Title/Abstract]) OR "acute kidney insufficiencies"[Title/Abstract] OR (("renal insufficiency"[MeSH Terms] OR ("Renal"[All Fields] AND "Insufficiency"[All Fields]) OR "renal insufficiency"[All Fields] OR ("Kidney"[All Fields] AND "Insufficiencies"[All Fields]) OR "kidney insufficiencies"[All Fields]) AND "Acute"[Title/Abstract]) | 85,777 |
| #9 | #7 OR #8 | 100,504 |
| #10 | "Mortality"[MeSH Terms] | 431,476 |
| #11 | "Mortality"[Title/Abstract] OR "Mortalities"[Title/Abstract] OR "mortality differential"[Title/Abstract] OR "mortality determinants"[Title/Abstract] OR "death rate"[Title/Abstract] OR "excess mortality"[Title/Abstract] OR "excess mortalities"[Title/Abstract] OR "mortality determinants"[Title/Abstract] OR "mortality determinant"[Title/Abstract] OR "case fatality rate"[Title/Abstract] OR "case fatality rates"[Title/Abstract] OR "Death"[Title/Abstract] | 1,837,018 |
| #12 | #10 OR #11 | 2,056,042 |
| #13 | #3 AND #6 AND #9 AND #12 | 78 |

**2.Embase**

| Search number | Query | Results |
| --- | --- | --- |
| #1 | 'machine learning'/exp | 524,402 |
| #2 | 'deep learning':ab,ti OR 'machine learning':ab,ti OR 'transfer learning':ab,ti OR 'prediction model':ab,ti OR 'artificial intelligence':ab,ti OR 'artificial neural network':ab,ti OR 'support vector machine':ab,ti OR 'random forest':ab,ti OR 'xgboost':ab,ti | 290,034 |
| #3 | #1 OR #2 | 594,188 |
| #4 | 'sepsis'/exp | 367,710 |
| #5 | 'Sepsis':ab,ti or 'Bloodstream Infection':ab,ti or 'Infection, Bloodstream':ab,ti or 'Septicemia':ab,ti or 'Septicemias':ab,ti or 'Blood Poisoning':ab,ti or 'Blood Poisonings':ab,ti or 'Poisoning, Blood':ab,ti or 'Severe Sepsis':ab,ti or 'Sepsis, Severe':ab,ti or 'Pyemia':ab,ti | 224,101 |
| #6 | #4 OR #5 | 421,949 |
| #7 | 'acute kidney failure'/exp | 137,877 |
| #8 | 'Acute Kidney Injury':ab,ti or 'Kidney Injuries, Acute':ab,ti or 'Kidney Injury, Acute':ab,ti or 'Acute Renal Injury':ab,ti or 'Acute Renal Injuries':ab,ti or 'Kidney Failure, Acute':ab,ti or 'Acute Kidney Failures':ab,ti or 'Kidney Failures, Acute':ab,ti or 'Acute Kidney Failure':ab,ti or 'Acute Renal Failure':ab,ti or 'Renal Insufficiency, Acute':ab,ti or 'Acute Renal Insufficiencies':ab,ti or 'Renal Insufficiencies, Acute':ab,ti or 'Acute Kidney Insufficiency':ab,ti or 'Acute Renal Insufficiency':ab,ti or 'Kidney Insufficiency, Acute':ab,ti or 'Acute Kidney Insufficiencies':ab,ti or 'Kidney Insufficiencies, Acute':ab,ti | 109,274 |
| #9 | #4 OR #5 | 159,968 |
| #10 | 'mortality'/exp | 524,402 |
| #11 | 'Mortality':ab,ti or 'Mortalities':ab,ti or 'Mortality, Differential':ab,ti or 'Mortality Determinants':ab,ti or 'Death Rate':ab,ti or 'Excess Mortality':ab,ti or 'Excess Mortalities':ab,ti or 'Mortality Determinants':ab,ti or 'Mortality Determinant':ab,ti or 'Case Fatality Rate':ab,ti or 'Case Fatality Rates':ab,ti or 'Death':ab,ti | 2,639,406 |
| #12 | #10 OR #11 | 3,063,992 |
| #13 | #3 AND #6 AND #9 AND #12 | 209 |

**3.Cochrane**

| Search number | Query | Results |
| --- | --- | --- |
| #1 | (Machine learning):ti,ab,kw OR (Deep Learning):ti,ab,kw OR (Deep Learning):ti,ab,kw OR (Transfer Learning):ti,ab,kw OR ( Prediction model):ti,ab,kw OR (Artificial intelligence):ti,ab,kw OR ( Artificial neural network):ti,ab,kw OR (Support vector machine):ti,ab,kw OR (Random forest):ti,ab,kw | 13,801 |
| #2 | (Sepsis):ti,ab,kw OR (Bloodstream Infection):ti,ab,kw OR (Infection, Bloodstream):ti,ab,kw OR (Septicemia):ti,ab,kw OR (Septicemias):ti,ab,kw OR (Blood Poisoning):ti,ab,kw OR (Blood Poisonings):ti,ab,kw OR (Poisoning, Blood):ti,ab,kw OR (Severe Sepsis):ti,ab,kw OR (Sepsis, Severe):ti,ab,kw OR (Pyemia):ti,ab,kw | 17,064 |
| #3 | (Acute Kidney Injury):ti,ab,kw OR (Kidney Injuries, Acute):ti,ab,kw OR (Kidney Injury, Acute):ti,ab,kw OR (Acute Renal Injury):ti,ab,kw OR (Acute Renal Injuries):ti,ab,kw OR (Kidney Failure, Acute):ti,ab,kw OR (Acute Kidney Failures):ti,ab,kw OR (Kidney Failures, Acute):ti,ab,kw OR (Acute Kidney Failure):ti,ab,kw OR (Acute Renal Failure):ti,ab,kw OR (Renal Insufficiency, Acute):ti,ab,kw OR (Acute Renal Insufficiencies):ti,ab,kw OR (Renal Insufficiencies, Acute):ti,ab,kw OR (Acute Kidney Insufficiency):ti,ab,kw OR (Acute Renal Insufficiency):ti,ab,kw OR (Kidney Insufficiency, Acute):ti,ab,kw OR (Acute Kidney Insufficiencies):ti,ab,kw OR (Kidney Insufficiencies, Acute):ti,ab,kw | 12,664 |
| #4 | #1 AND #2 AND #3 | 19 |

**4.Web of science**

| Search number | Query | Results |
| --- | --- | --- |
| #1 | TS=(Machine learning or Deep Learning or Transfer Learning or Prediction model or Artificial intelligence or Artificial neural network or Support vector machine or Random forest or XGboost) | [142,494](https://www.webofscience.com/wos/woscc/summary/94af2a60-8e33-439f-905b-9b06fb6dc874-01057f7b83/relevance/1) |
| #2 | TS=(Sepsis or Bloodstream Infection or Infection, Bloodstream or Septicemia or Septicemias or Blood Poisoning or Blood Poisonings or Poisoning, Blood or Severe Sepsis or Sepsis, Severe or Pyemia ) | [4,632](https://www.webofscience.com/wos/woscc/summary/50887b8b-da9d-4c9a-9d05-cbab8bc67b9e-01057f91fc/relevance/1) |
| #3 | TS=(Acute Kidney Injury or Kidney Injuries, Acute or Kidney Injury, Acute or Acute Renal Injury or Acute Renal Injuries or Kidney Failure, Acute or Acute Kidney Failures or Kidney Failures, Acute or Acute Kidney Failure or Acute Renal Failure or Renal Insufficiency, Acute or Acute Renal Insufficiencies or Renal Insufficiencies, Acute or Acute Kidney Insufficiency or Acute Renal Insufficiency or Kidney Insufficiency, Acute or Acute Kidney Insufficiencies or Kidney Insufficiencies, Acute ) | 1,422 |
| #4 | TS=(mortality or Mortalities or Mortality, Differential or Mortality Determinants or Death Rate or Excess Mortality or Excess Mortalities or Mortality Determinants or Mortality Determinant or Case Fatality Rate or Case Fatality Rates ) | [187,934](https://www.webofscience.com/wos/woscc/summary/42289e28-9a12-4de6-9513-e6fe541b9467-01057fa6ba/relevance/1) |
| #4 | #1 AND #2 AND #3 AND #4 | 6 |
