# Supplementary material for: Machine learning for the prediction of mortality in patients with sepsis-associated acute kidney injury: a systematic review and meta-analysis
Source: BMC Infect Dis. 2024 Dec 21;24:1454. doi: 10.1186/s12879-024-10380-6 (PMC11663330; doi:10.1186/s12879-024-10380-6)
Supplement: Supplementary file 3 — Supplementary Material 3: Detailed information on model performance [file 12879_2024_10380_MOESM3_ESM.docx]

| **author** | **ML algorithm** | **Model performance** | | | | | | |
| --- | --- | --- | --- | --- | --- | --- | --- | --- |
|  |  | **Train set C index**  **(95%CI)** | **Test set C index (95%CI)** | **Sensitivity (95%CI)** | **Specificity (%)** | **Accuracy (%)** | **Average precision** | **F1 Score** |
| Lei Dong | LR | 0.839 | 0.749 | - | - | - | - | - |
|  | Lasso | 0.832 | 0.729 | - | - | - | - | - |
|  | Rpart | 0.747 | 0.604 | - | - | - | - | - |
|  | RF | 0.875 | 0.644 | - | - | - | - | - |
|  | XGBoost | 0.936（0.932-0.941） | 0.796 | - | - | - | - | 0.743 |
|  | ANN | 0.867 | 0.743 | - | - | - | - | - |
| Zhiyan Fan | LR | 0.740(0.720-0.760） | 0.670（0.650-0.690） | - | - | - | 0.692 | 0.692 |
|  | RF | 0.790(0.770-0.810） | 0.670（0.650-0.690） | - | - | - | 0.719 | 0.719 |
|  | XGBoost | 0.830(0.810-0.850） | 0.790（0.770-0.810） | - | - | - | 0.765 | 0.765 |
|  | MLP | 0.750(0.730-0.770） | 0.730（0.710-0.750） | - | - | - | 0.684 | 0.683 |
|  | SVC | 0.760(0.740-0.780） | 0.690（0.670-0.710） | - | - | - | 0.699 | 0.700 |
| Tianyun Gao | LR | 0.756(0.732-0.779) | 0.790 | - | - | 0.816 | 0.569 | 0.258 |
|  | SVM | 0.718(0.691-0.743) | 0.690 | - | - | 0.809 | 1.000 | 0.008 |
|  | NB | 0.764(0.741-0.786) | 0.780 | - | - | 0.795 | 0.446 | 0.354 |
|  | XGBoost | 0.796(0.774-0.817) | 0.810 | - | - | 0.823 | 0.569 | 0.414 |
|  | RF | 0.798(0.774-0.821) | 0.820 | - | - | 0.832 | 0.661 | 0.372 |
|  | KNN | 0.689(0.663-0.714) | 0.690 | - | - | 0.805 | 0.487 | 0.359 |
|  | DT | 0.635(0.611-0.659) | 0.640 | - | - | 0.765 | 0.395 | 0.409 |
| Xunliang Li | LR | 0.730 (0.694–0.765) | 0.739(0.694-0.765) | 0.608 | 0.754 | 0.822 | 0.572 | - |
|  | SVM | 0.680 (0.643–0.717) | 0.680(0.643-0.717） | 0.562 | 0.736 | 0.826 | 0.556 | - |
|  | KNN | 0.601 (0.563–0.638) | 0.601(0.563-0.638) | 0.367 | 0.783 | 0.793 | 0.429 | - |
|  | DT | 0.585 (0.547–0.623) | 0.580(0.547-0.623) | 0.378 | 0.812 | 0.737 | 0.425 | - |
|  | RF | 0.778 (0.745–0.812) | 0.778(0.745-0.812) | 0.739 | 0.674 | 0.825 | 0.622 | - |
|  | XGBoost | 0.794 (0.762–0.827) | 0.794(0.762-0.827) | 0.793 | 0.752 | 0.832 | 0.660 | - |
| Xiaoqin Luo | XGBoost | 0.804(0.798-0.81) | 0.748(0.742-0.753) | 80.1 | 0.729 | - | - | - |
|  | RF | 0.798(0.792-0.804) | 0.745(0.740-0.751) | - | - | - | - | - |
|  | SVM | 0.767(0.760-0.773) | 0.720(0.715-0.726) | - | - | - | - | - |
| Jie Tang | LR | 0.791 | 0.771 | 0.25 (0.22, 0.27) | - | - | - | 0.355 |
|  | SVM | 0.788 | 0.764 | 0.25 (0.23, 0.28) | - | - | - | 0.368 |
|  | GBM | 0.856 | 0.802 | 0.40 (0.37, 0.43) | - | - | - | 0.526 |
|  | AdaBoost | 0.917 | 0.750 | 0.56 (0.53, 0.58) | - | - | - | 0.646 |
|  | XGBoost | 0.836 | 0.799 | 0.35 (0.32, 0.37) | - | - | - | 0.462 |
|  | CatBoost | 0.844 | 0.804 | 0.36 (0.34, 0.39) | - | - | - | 0.489 |
|  | NB | 0.803 | 0.793 | 0.27 (0.25, 0.30) | - | - | - | 0.388 |
|  | NN | 0.812 | 0.789 | 0.34 (0.31, 0.36) | - | - | - | 0.440 |
|  | MLP | 0.814 | 0.792 | 0.38 (0.35, 0.40) | - | - | - | 0.472 |
|  | KNN | 0.825 | 0.787 | 0.26 (0.24, 0.28) | - | - | - | 0.382 |
|  | RF | 0.987 | 0.795 | 0.63 (0.61, 0.66) | - | - | - | 0.764 |
| Jijun Yang | XGBoost | 0.873(0.86-0.886) | - | - | 0.973 | 0.773 | 0.724 | 0.801 |
|  | GBM | 0.865(0.851-0.878) | - | - | 0.865 | - | - | - |
|  | RF | 0.849(0.834-0.863) | - | - | 0.849 | - | - | - |
|  | LR | 0.850(0.836-0.864) | - | - | 0.850 | - | - | - |
| Hongshan Zhou | CatBoost | 0.827 | 0.754 | 0.750 | - | 0.750 | - | 0.560 |
|  | GBDT | 0.823 | 0.624 | 0.790 | - | 0.710 | - | 0.530 |
|  | LightGBM | 0.819 | 0.612 | 0.750 | - | 0.740 | - | 0.550 |
|  | AdaBoost | 0.819 | 0.595 | 0.650 | - | 0.790 | - | 0.570 |
|  | RF | 0.818 | 0.631 | 0.660 | - | 0.780 | - | 0.550 |
|  | XGBoost | 0.812 | 0.574 | 0.680 | - | 0.770 | - | 0.550 |
|  | KNN | 0.801 | 0.631 | 0.730 | - | 0.720 | - | 0.520 |
|  | MLP | 0.793 | 0.632 | 0.700 | - | 0.730 | - | 0.520 |
|  | LR | 0.788 | 0.709 | 0.710 | - | 0.730 | - | 0.520 |
|  | NB | 0.763 | 0.602 | 0.740 | - | 0.680 | - | 0.490 |
|  | SVM | 0.761 | 0.679 | 0.690 | - | 0.740 | - | 0.530 |
